# Human Pangenomics: Promises and Challenges of a Distributed Genomic Reference

**DOI:** 10.3390/life13061360

**Published:** 2023-06-09

**Authors:** Paolo Abondio, Elisabetta Cilli, Donata Luiselli

**Affiliations:** Laboratory of Ancient DNA, Department of Cultural Heritage, University of Bologna, Via degli Ariani 1, 48121 Ravenna, Italy

**Keywords:** pangenomics, human genomics, pangenome, structural variation, bioinformatics, evolution, selection, phylogenetics, public health, personalized medicine

## Abstract

A pangenome is a collection of the common and unique genomes that are present in a given species. It combines the genetic information of all the genomes sampled, resulting in a large and diverse range of genetic material. Pangenomic analysis offers several advantages compared to traditional genomic research. For example, a pangenome is not bound by the physical constraints of a single genome, so it can capture more genetic variability. Thanks to the introduction of the concept of pangenome, it is possible to use exceedingly detailed sequence data to study the evolutionary history of two different species, or how populations within a species differ genetically. In the wake of the Human Pangenome Project, this review aims at discussing the advantages of the pangenome around human genetic variation, which are then framed around how pangenomic data can inform population genetics, phylogenetics, and public health policy by providing insights into the genetic basis of diseases or determining personalized treatments, targeting the specific genetic profile of an individual. Moreover, technical limitations, ethical concerns, and legal considerations are discussed.

## 1. Introduction

The concept of pangenome (or “pan-genome”, also called “supragenome”) traces its origin in the molecular study of Prokaryotes, as it mostly refers to the entirety of the transcribed genetic units in all available lineages of a monophyletic (bacterial) group [1,2,3]. Gathering all the genomes of a phylogenetic clade would provide the result of assembling all the genes for that group in a superior genetic structure that could encompass its complete genetic diversity and repertoire in a theoretical object called a “supergenome” [4,5]. Therefore, the pangenome represents the total compendium of all possible genomic variability for a collection of specimens ideally sharing a common ancestor [6], but does not describe the complete genetic makeup of a species, which could only be achieved by sequencing every single genome of that species. Of course, this definition, although fitting, is influenced by the very nature of the average bacterial genome and the way genetic material can be exchanged among unicellular Prokaryotes. Indeed, the bacterial genome is frequently small, haploid, and almost devoid of introns or intergenic DNA, yet its composition in terms of transcriptional units is extremely flexible, as only a handful of genes are absolutely crucial for the actual functioning of the bacterium, while a wider cluster of accessory genes, mediating survival in specific environmental conditions and other collateral properties (for example, antimicrobial resistance or toxin production), may be integrated, lost, and exchanged through horizontal transfer, or picked up from the environment as fragments of free-floating DNA [7,8,9]. So, what guarantees rapid adaptation and evolution, as well as extreme plasticity, is the existence of this flexible genomic content that is characteristic of Prokaryotes and that constitutes the “pangenome”.

On the other hand, multicellular Eukaryotic organisms usually contain multiple fragments of DNA (chromosomes) as well as multiple copies of the same DNA strand in each cellular nucleus (polyploidy) [10,11]. These multiple copies carry similar coding information, and virtually all possible genes are always present (although not all actively functioning) in the genetic sequences of each single individual. However, the intricacy of Eukaryotic DNA in complex multicellular organisms lies in it being highly enriched in non-genic material and characterized by a higher degree of structural variation, which moves way beyond individual differences in single nucleotide polymorphisms [12,13,14]. The notion of pangenome, then, requires the inclusion of novel genetic compositions, whereby its properties are expanded and modified to accommodate a wider and much more complex definition. Indeed, a more general depiction should acknowledge that genomic variation encompasses point mutations but also insertions/deletions, repetitive DNA, mobile genetic elements, inversions, duplications, and gene fusions, especially since gene presence/absence cannot be an informative descriptor of genomic variability in high-order groupings of Eukaryotes [15,16]. Similarly, the “population” under scrutiny can be a taxonomic unit [14,17] but can also be a collection of cells from the same tissue [18] or an ecological community [19,20] depending on what level of biological description one is trying to analyze. Moreover, it must be taken into consideration that many different processes take place having the genetic code on the background, and that these are much more variable among cells and individuals than the simple DNA sequence. Epigenetic patterns [21] and cell-specific chromatin remodeling [22], leading to regulation of transcription and differential translation [23,24], are only some of the phenomena that intervene to determine the flow of information towards the expression of the genetic code, and these can also be considered and studied in a similar way to the pangenome (pan-epigenomics [25,26], pan-transcriptomics [27], pan-proteomics [28], and so on) to encapsulate the overall diversity and general variability of genomic products in a population. Quite recently, the potential of a pangenomic approach (i.e., the identification of gene clusters and definition of relationships between genomes based on gene sharing) has also been applied in the context of metagenomics (i.e., the ability to sequence microbial DNA directly from the environment and define phylogenetic relationships across the spatial dimension of ecological niches) to describe the ecological role of gene clusters linked to niche adaptation and fitness in microbial clades, giving rise to the discipline of pan-metagenomics [29,30,31,32,33].

## 2. Characteristics of the Pangenome

**Figure 1:**
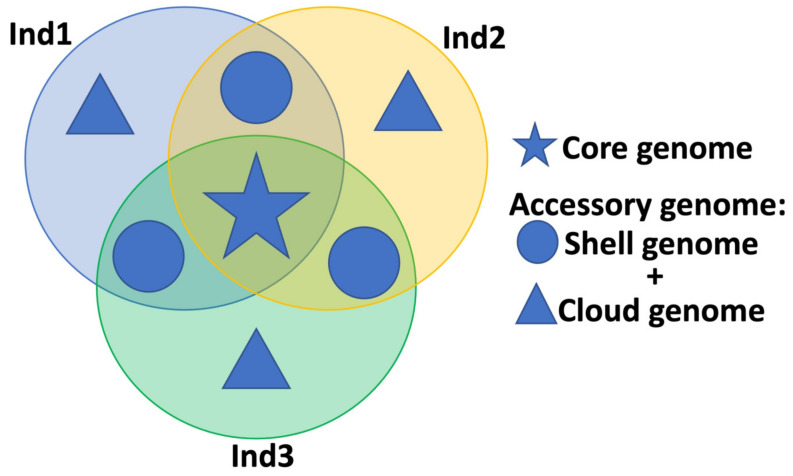
Example of core and accessory genome from three sequenced samples.

The pangenome concept, as introduced in Section 1, can be applied to any biological population, be it a viral, bacterial, or eukaryotic species. A pangenome can be considered as a collection of all the genes, regulatory entities, and non-genetic segments that are present in different numbers in various lineages of the group under scrutiny. It is also possible to identify which genes are not part of the core collection and which are essential—that is, those that are found in all or most of the lineages (Figure 1). The core genes are conserved, while the accessory genes, present in only some individuals or lineages, are more variable [3,5,34,35]. Moreover, genetic segments that are part of the accessory genome can be further subdivided in the “dispensable” or “shell” genome (i.e., structures that are shared by at least two subjects) and the “unique” or “cloud” genome (i.e., the collection of genomic elements that are specific to a single individual). Sometimes, the definition of “dispensable” genome also encompasses the unique genome, coinciding in this case with the accessory genome [17]. This allows for the characterization of the species’ genetic diversity and for the identification of new alleles and their potential impact on the species’ microevolution [6,36,37]. By comparing the pangenomes of two species, in fact, it is possible to identify which genes are shared and which are unique; this can also provide insights into the evolutionary history of the species and their relationship with each other [38,39]. The pangenome is also an important concept for phylogenetic studies; by comparing the pangenomes of different species, it is possible to identify the evolutionary relationships between them. This can be used to resolve the affiliations between different species and to study the evolution of new genetic structures [4,40,41]. Finally, the pangenome concept can be applied to the study of the genetic basis of adaptation; by comparing the pangenomes of different species, it is possible to identify the genes that are associated with peculiar phenotypes and to understand how they are related [1,36]. This can provide insights into how particular traits have evolved and how they are maintained across different taxonomic units.

The pangenome concept can be formalized by focusing on its different components, which aim to provide a better understanding of the structure and dynamics of the gene content of a population. It can be tied to notions of static and dynamic genome composition and evolution, each of which has its own advantages and limitations. Static formulations of the pangenome can analyze the gene content of a population at a given time or location and are typically used to describe the core and accessory components of the pangenome [42,43]. A focus on core genes, for instance, considers only those that are shared by all members of the population and disregards the variability of the accessory genome. This model is useful for framing the stability and resilience of a population in changing environmental conditions by identifying shared or conserved genomic elements and can be used to pinpoint common druggable targets for antibiotic therapy [42,44,45]. An expanded model, on the other hand, may consider the variability of the accessory genome and provide a more accurate description of the genetic repertoire of a species, highlighting the emergence of novel genes by selection and adaptation [46]. The pangenome, on the other hand, can also be considered in its dynamic structure, by analyzing the temporal changes in the gene content of a population [8]. A “gene birth-and-death” model [47], for example, could consider the acquisition and loss of genes along a phylogeny and over time in order to predict the emergence of novel genetic components and, consequently, the evolutionary dynamics of a population [33,48]. However, they also have their own limitations, such as the fact that they may not consider stochastic changes in gene frequencies over time, the effects of gene interactions, and the complex regulatory networks that control the expression of genes in a population.

## 3. The Paradigm of Human Cell Types as Species

The concept of pangenome has recently been extended to higher-order organisms, and is being applied most notably to the human species [49,50,51,52]. By extension, the notion of a pangenome is based on the idea that different cell types can be regarded as “species” in their own right, each with a unique constitution of genetic material [18,53]. Indeed, human cells differ from each other in terms of their transcriptomes, proteomes, and metabolomes, as well as their genomic content [21,54,55]. This means that a single human tissue can contain several different genetic programmes, and this heterogeneity is reflected in the different collections of cell types that exist, as in the case of immune T cells studied at single-cell level [54,56,57]. This view allows researchers to study the genetic composition of the same individual at different levels of detail, from the single cell to the entire organism [58,59]. Pangenome analysis of human cells can therefore be used to identify genetic differences between cell types, as well as to identify genes that are associated with particular cellular phenotypes. Such analysis can help us to understand the function of individual genes, as well as to identify novel gene products that are associated with specific cell types. Additionally, pangenome analysis can also be used to identify genetic elements that are associated with diseases, such as cancer, and to develop therapeutic approaches that target these objects [60,61]. Indeed, although some cancers present a non-negligible familiarity due to germline mutations in specific target genes [62,63,64], most of them are somatic in nature, and a pangenomic approach to their cellular makeup has become a primary way of exploring the molecular identity and phenotypic variability of this relatively common pathology [53,60,61,65,66].

So, the pangenome provides a powerful tool for understanding the complexity of human cells and for developing novel therapies for the treatment of different human diseases. This concept is particularly relevant to the field of personalized medicine, as it allows for a much more targeted approach to diagnosis and treatment. By considering the individual’s genetic composition at different levels, a better assessment could be provided for the likelihood of a patient developing a particular condition, or how well a particular treatment will work for them. In addition, the concept of pangenome has important implications for the field of biotechnology. By understanding the pangenomic composition of different cell types, it is possible to develop new technologies that are tailored to work with a particular set of genetic material. For example, gene-editing techniques such as CRISPR-Cas9 can be used to modify the genetic material of a particular cell type in order to perform lab screening, treat a particular condition, or to create a new type of cell with desired characteristics [67,68]. This has the potential to revolutionize the way targeted treatments are designed and applied in a clinical setting.

## 4. Describing the Repertoire of Structural Variation

Structural variation (SV) refers to the large-scale changes in the structure of the genome, such as deletions, duplications, inversions, and translocations [69]. These changes can occur in both coding and non-coding regions and can lead to significant changes in gene expression, function, and underlying phenotype [70,71,72,73]. SVs are a major contributor to the diversity of the human genome, and they can cause disease when they alter important regulatory elements or disrupt essential genes [74,75]. The vast majority of SVs are not found in the reference genome, so their detection and characterization are challenging [76,77,78,79]. The study of structural variation has been greatly facilitated by the development of high-throughput sequencing technologies, as these allow for the detection of such events at a much higher resolution and in larger numbers than previously possible [76,80]. The repertoire of SVs that can be found in a given population is vast, and it is constantly evolving; SVs are dynamic and can be used by a variety of processes, such as the unequal crossing over between homologous chromosomes, non-homologous end joining, and replication errors; they can also be inherited from parent to offspring or can be acquired de novo; they can also occur in any region of the genome, from the smallest single nucleotide changes to the largest chromosomal rearrangements [81,82,83]. The study of SVs provides important insights into the evolution of the human genome, as well as the genetic basis of diseases, as they can affect gene expression, leading to changes in phenotype, and can influence the efficacy of certain treatments [82,83]. In addition, SVs can provide important clues to the evolutionary history of a species, as they can provide evidence of recombination events between closely related lineages or between populations [79,83,84]. As such, SVs are an important aspect of the pan-genome, providing a dynamic view of the evolution of a species over time.

## 5. Increasing SNP Discovery, Mappability, and Association

Recent advances in the field of genomics have facilitated the identification of single nucleotide polymorphisms (SNPs), which are the most common type of genetic variation in the human genome [85,86,87]. SNPs are small genetic changes that can occur at a single base pair and, when they occur within a gene or a regulatory region of the genome, they can affect its function or the regulation of its expression [88,89,90]. While the discovery of SNPs is relatively straightforward, the challenge lies in their mapping and in determining how they contribute to the phenotype of an individual [91]. The ability to map SNPs has been greatly enhanced by the development of high-throughput sequencing technologies such as next-generation sequencing (NGS), which allows for rapid and cost-effective whole-genome sequencing. The NGS data generated can then be used to identify SNPs and map them to specific locations in the genome. These data can also be used to identify gene expression patterns associated with SNPs and to determine how they contribute to phenotypic variation. In addition to advances in technology, the development of databases and bioinformatics tools has also enabled the search for SNPs in large datasets. The use of bioinformatics tools to integrate SNP data with other types of data, such as transcriptomic and proteomic data, has also enabled researchers to identify correlations between SNPs and phenotypic variation [92,93,94]. This has been particularly useful in the field of personalized medicine, where the identification of SNPs associated with disease can help to develop more precise treatments and therapies [95,96]. The pangenome has enabled a much better understanding of genetic variation and diversity among organisms, allowing for an unprecedented level of SNP discovery and mappability [97,98]. Indeed, similarly to structural variants, by studying the entire genomic content of a population rather than individual genomes, a much wider range of SNPs can be identified. The pangenome can be used to improve the accuracy of existing genetic maps, as it can be used to infer population structure and identify recombination hotspots between different lineages [99,100]. This can then be used to refine existing maps and identify areas of higher recombination, which is especially important for genome-wide association analyses. Conversely, the pangenome can also be used to identify areas of conserved genetic variation, which can aid in the identification of regions of interest in evolutionary studies [98]. Finally, the pangenome can also be used to create more accurate and comprehensive databases of SNPs, which can then be used to facilitate the development of more powerful bioinformatics tools and data mining techniques [101,102,103]. So, by utilizing the pangenome, researchers can compare the entire genome of an individual to the various genetic compositions present within a population and identify SNPs that are specific to an individual. This allows for a more comprehensive analysis of the effects of an SNP on the genetic code of an individual, as well as providing a more detailed understanding of the population-specific genetic makeup.

In the context of SNP-phenotype relationship elucidation, genome-wide association studies (GWAS) have been increasingly important for deciphering the complex relationships between genes and physical characteristics [104,105,106,107]. GWAS is a type of statistical approach that seeks to identify correlations between genetic variants in large populations starting from given phenotypes [108]. Initially undertaken with the application of single nucleotide polymorphisms (SNPs), GWAS approaches have since been adapted for use with other genetic variants such as insertions and deletions [108]. An important consideration in the effectiveness of GWAS is the sample size used, as the larger the sample, the more accurate and comprehensive the results become [109,110,111]. The use of pangenomics as part of GWAS has grown in recent years as a means of improving the accuracy of estimated associations (pan-GWAS) as well as novel discovery [14,112,113,114]. Indeed, the collective term “pangenomics” includes different types of data and information that are taken from multiple sources of genetic material through whole-genome sequencing and genotyping. From these datasets, an extended set of SNPs and other variants can be identified, yielding more genetic information than is available with single-source datasets. Therefore, using pangenomics-based genetic data in GWAS, researchers can obtain more precise and comprehensive understanding of the genetic variants associated with diseases and other phenotypes at a higher level of taxonomic description. Interestingly, the use of pangenomics has also extended to the study of the roles of gene-gene (or eQTL) interactions in regulating gene expression levels and the resulting phenotypes [114,115]. In this way, pangenomics can directly enhance GWAS results, as the genome-wide scan can be augmented with additional data on eQTLs and protein–protein interactions to more accurately identify potentially causative genetic variants which may be involved in complex multifactorial diseases such as diabetes or dementia. Another useful tool in pangenomic-driven association studies is cell-line (or organoid [116,117,118])-based phenotypic screening [119,120]. This technique involves using cell-based models, such as a lab-created in vitro cell cultures, to screen for a specific phenotype change in response to a genetic variant. By introducing an engineered genetic variant detected through pan-GWAS into a cell, researchers can use high-throughput assays to assess the effect of the variant on a particular phenotype. This approach, supplementing genome-wide scans, provides results which can be directly attributed to a genetic variant.

Overall, the pangenome concept represents a powerful tool for increasing the discovery and resolution of single nucleotide polymorphisms (SNPs), as well as structural variants, in the human genome. By providing a more comprehensive analysis of the genetic code of an individual, as well as providing insights into the population-specific genetic makeup, the pangenome is a valuable tool for researchers studying the genetic diversity of human populations.

## 6. Pangenomic Non-Linearity and Larger Structural Variations

**Figure 2:**
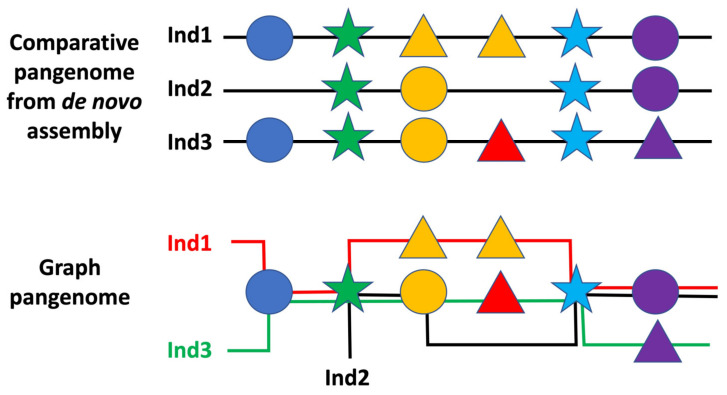
Example of a comparative and a graph interpretation of the pangenome based on three sequenced individuals. Symbols correspond to genomic elements belonging to the core, shell or cloud genome as shown in Figure 1. Colors represent elements in the same position. Individual 1 (Ind1) shows a duplication of the third genomic element, which is different than the one carried by the other individuals and is, therefore, part of the cloud genome.

The concept of the pangenome has opened up the possibility of looking into the genome as a non-linear object (Figure 2). This is because the genomes of individuals are not necessarily identical, and different individuals may have different genomic compositions [121]. In fact, there are various factors that influence the genetic makeup of individuals, such as genetic recombination, mutations, and even environmental factors [122,123], and this means that the genetic composition of individuals can vary, even though they are members of the same species. As such, the study of the human pangenome has the opportunity to become a powerful tool for further understanding the complexity of genome diversity of humans, i.e., their structural variation between individuals, aiming for better understand the genetic basis of common diseases. Additionally, the study of the human pangenome can also help us to better disentangle the evolution of the human genome, and how different genetic components interact with one another. The implications of the human pangenome go beyond just the study of disease, as it can also be used to explore the functional roles of different genes and gene clusters in various biological processes and how they interact with one another in various ways [124,125,126]. Thinking in terms of population genetics, the fact that the pangenome can be interpreted as a non-linear object implies that the genetic potential of a group of individuals is not restricted to a linear arrangement of nucleotides, but instead involves a much more complex repertoire of structural variation that may be shaped at the individual level by various epigenetic, regulatory, and metabolic patterns. Furthermore, it is now known that processes such as gene expression and chromatin remodelling are largely responsible for drastically altering the quality, quantity, and even the type of gene products generated from the same sequence of DNA. This means that the same gene in the pangenome can give rise to different outcomes, depending on how it is regulated and expressed, implying that the linearity of a single individual’s DNA is not enough to determine the final outcome of a particular gene. In addition, pangenomic non-linearity also allows for the detection of novel trait-related structural gene variants that may not have been possible to identify before, similarly to the accessory genes that favour the development of drug resistance in bacteria or the emergence of new morphological features in plants. Ideally, the non-linearity of the pangenome through structural variation is an essential factor that may contribute to the evolution of a species and the development of novel traits. It is also relevant to the study of human health and disease, as it can help to explain the diversity of clinical manifestations seen in various genetic disorders and can assist in the development of better therapies and treatments.

## 7. Technical, Ethical, and Legal Considerations

The prospect of a human pangenome holds tremendous potential for applications ranging from personalized medicine to forensics and population genetics. However, the technical implications of such a project should not be overlooked [49,50]. Indeed, the sheer amount of data that would need to be generated and stored to map the human pangenome is immense and could prove to be an obstacle for its implementation. For example, the number of individuals that would need to be genotyped in order to obtain a reliable and accurate representation of the human pangenome could be so large that it is not necessarily feasible to do it with the current technology. However, novel bioinformatic methods have been developed in recent years to allow the analysis of such mass of data from microbes, plants, and animals [98,127,128,129]. Furthermore, the data generated from individuals involved in such projects would not only be massive, but also highly sensitive and personal, as they would contain the complete and exact sequence of an individual’s genetic material [130,131,132,133,134]. This raises ethical issues related to the right to privacy and data protection, as well as potential misuse, as the data could be used to identify subjects or to discriminate against certain populations or individuals [135,136,137,138,139]. Human genome analysis is already being used to identify new ways to diagnose and treat medical conditions, as well as to better understand the underlying genetic basis of disease. By understanding a person’s full genetic makeup, doctors can more accurately diagnose diseases, as well as tailor treatments to target specific genetic mutations. So, the pangenome can be used to develop personalized medicine, where treatments are tailored to an individual’s unique genetic profile. Overall, the pangenome has the potential to revolutionize genetics-informed medical practice and provide valuable insight into the genetic basis of diseases. On the other hand, the use of the pangenome could also lead to further ethical dilemmas. For example, it could potentially be used to discriminate against individuals or communities based on their genetic makeup. This could lead to people being denied access to healthcare, education, or employment, due to their genetic profile [140,141]. It is also important to consider the potential repercussions of data privacy and sharing around the human pangenome in order to ensure that it is used responsibly and ethically. For example, who owns and has the right to access the data that is generated? Who has the right to determine how the data should be used? How will the data be used and how will they be protected from misuse? What legal framework can be put in place to ensure that the data are used in a responsible and appropriate way [142,143,144,145]? From a public health perspective, it is possible that the data generated could reveal important information about certain diseases or health conditions or could be used to predict certain health outcomes. As such, it is important to consider how this information should be used, as well as the potential implications for public health policy [146,147,148]. If a person’s entire genetic make-up were to be known, then it would be possible to identify and compare them to others with similar genetic profiles, with legal issues concerning, in particular, privacy and the unauthorized use of genetic data; an individual’s genetic profile could be used to determine whether they may be predisposed to certain diseases or conditions, and this information could be used to deny them rights or opportunities. This could be especially concerning if employers or insurance companies were to gain access to an individual’s genetic profile and use it to deprive them of access to a job or health insurance [149]. In addition, the privacy of an individual’s genetic information could be compromised if it were to be shared without their knowledge or consent. Furthermore, the use of the human pangenome could also lead to further legal issues in the field of intellectual property. For instance, if a gene or genes were to be identified as being responsible for a particular trait, then it might be possible to patent those genes [150,151,152,153]. This could potentially lead to disagreements over who has the rights to the gene and its uses. Furthermore, the infringement of such patents could also potentially lead to further legal disputes. Finally, the use of the pangenome (both human and microbial) could also lead to further issues in the field of criminal justice [154,155]. For instance, it could potentially be used for suspect identification, and this could possibly lead to legal debates over the accuracy and reliability of the evidence. In addition, the use of genetic information could also potentially lead to arguments over the ethical and moral implications of using such evidence in criminal trials. Overall, the legal implications of the human pangenome are complex and far-reaching. Although the prospective benefits of utilizing this information are great, the potential risks and implications must also be taken into consideration. The concerns emerging from the use, production, and discovery of genetic data are, of course, already known at the level of the single individual; therefore, it is essential that any legal framework surrounding the use of this information be carefully considered and that appropriate measures are taken to ensure the privacy, safety, and ethical use of such data at a population level as well. Indeed, legal and technical safeguards are already in place to protect not only the physical entity that is the DNA but also the flow of all derived genomic data information, as well as to uphold anonymity and confidentiality, such as via cryptography, access control, and data perturbation [156,157,158]. However, in a world where at-home genetic testing is easily accessible for a small fee, and consumer genetics companies are allowed to sell their data to pharmaceutical corporations for drug development [159,160,161] or provide them to law enforcement [162,163], moving through the legal and ethical maze of what constitutes explicit consent to data sharing is still very unsteady territory and must be thoroughly scrutinized [164,165,166]. As subject privacy and data sharing cannot be mutually exclusive in the setting of a truly democratic science, encrypting the individual’s genetic data but providing access to the pangenome, which does not inform on any single person’s specific characteristics, may seem like a reasonable trade-off.

## Data Availability

Not applicable.
